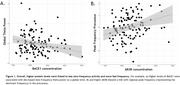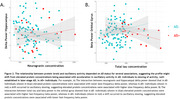# Proteins involved in amyloid metabolism and synaptic integrity are associated with neurophysiological changes in cognitively normal elderly individuals: an MEG study

**DOI:** 10.1002/alz70856_104945

**Published:** 2026-01-07

**Authors:** Senne B Lageman

**Affiliations:** ^1^ Alzheimer Center Amsterdam, Neurology, Vrije Universiteit Amsterdam, Amsterdam UMC location VUmc, Amsterdam, North Holland, Netherlands

## Abstract

**Background:**

In Alzheimer's disease (AD), amyloid‐beta (Aß) pathology induces neuronal hyperactivity when cognition is still normal. Although this effect on a macroscale is difficult to detect non‐invasively and accurately in patients, alterations in brain activity can be detected using magnetoencephalography (MEG) in preclinical AD. Here, we tested the hypothesis that proteins involved in amyloid metabolism and synaptic integrity contribute to changes in brain activity in preclinical AD.

**Method:**

This study included 120 cognitively normal (CN) older individuals who underwent cognitive testing, resting‐state MEG, a lumbar puncture to obtain cerebrospinal fluid (CSF) and a [^18^F]flutemetamol PET scan to determine Aß (+/‐) status. MEG spectral relative power was calculated globally and regionally (hippocampus, precuneus and orbital gyrus) in the canonical frequency bands, as was peak frequency. AD‐related proteins involved in amyloid metabolism and synaptic integrity were measured in CSF, including Aß38, BACE1, neurogranin and total tau. Linear regression models were employed to investigate associations between spectral power and protein concentrations. Interaction effects were calculated to test the dependance of these associations on Aß status.

**Result:**

Overall, we observed that higher protein levels were linked to acceleration of oscillatory activity (Figure 1). Higher BACE1 concentrations were associated with lower global and regional theta power and higher regional alpha2 power, higher t‐tau levels were associated with lower hippocampal delta power and higher Aß38 levels were related to higher peak frequency in the precuneus. Finally, we observed that the relationship between protein levels and delta power in the hippocampus depended on Aß status, as was the case for the delta, theta and beta power in the orbital gyrus, suggesting that in Aß+ individuals higher protein levels were associated with oscillatory slowing (Figure 2).

**Conclusion:**

Our findings indicate that proteins involved in amyloid metabolism and synaptic integrity are associated with acceleration in global and regional oscillatory power in CN adults. This profile might already shift from acceleration in oscillatory activity in Aß‐ individuals to slowing of activity, well‐established in later‐stage AD, in Aß+ individuals. Our results offer novel insights into the relationship between pathology and brain activity and provide new support for brain activity as an early biomarker.